# Natural Manganese Ore as a Functional Additive in Calcium Aluminosilicate Glasses

**DOI:** 10.1021/acsomega.6c02999

**Published:** 2026-08-21

**Authors:** José G. O. Silva, Rai F. Jucá, Israel F. Cardoso, Adriana P. B. dos Santos, João M. Soares

**Affiliations:** † Department of Physics, 89113 State University of Rio Grande do Norte, Mossoró, Rio Grande do Norte 59610-210, Brazil; ‡ Department of Physics, 67843 Federal University of Ceará, Fortaleza, Ceará 60021-970, Brazil; § Department of Chemistry, 89113 State University of Rio Grande do Norte, Mossoró, Rio Grande do Norte 59610-210, Brazil

## Abstract

We report the influence of manganese ore addition on the structural, thermal, optical, and magnetic properties of calcium aluminosilicate glasses synthesized using scheelite tailings as the main precursor, doped with different amounts of natural manganese ore (1–10 wt %). The samples were prepared by the melt-quenching method at 1400 °C in a graphite crucible followed by rapid cooling. The elemental composition confirmed the increase of Mn content with ore addition. X-ray diffraction revealed a broad diffuse scattering characteristic of an amorphous structure, confirming the formation of a glassy matrix. Transmission electron microscopy indicated the presence of amorphous nanoparticles or nanoclusters dispersed within the matrix, with more pronounced clustering at higher Mn concentrations. Infrared spectroscopy revealed structural modifications in the silicate network, including shifts in the Si–O–Si and Si–O–Al vibrational bands and the increase of nonbridging oxygen sites, suggesting that manganese promotes structural rearrangements and partial depolymerization of the glass network, consistent with a modifier-like behavior. Thermal analysis revealed glass transition temperatures between 781 and 809 °C and crystallization temperatures between 1032 and 1116 °C, indicating good thermal stability. The optical results show the transmittance and absorbance curves and the electronic transitions, explaining the color variation among the samples and providing the energy level for the electronic states using Racah parameters. The estimated band gap for the samples resulted in values between 1.75 and 2.05 eV, except for the least Mn-incorporated sample (not determined). The magnetic results show *g*-factors around 2.0, corresponding to Fe^3+^ and Mn^2+^ signals and an enlargement of the peak-to-peak line width (Δ*H*_*pp*_), indicating the presence of clusters or particles immersed in the glass matrix. Hysteresis measurements presented an increase in magnetization and coercivity with manganese ore increase, resulting from the Mn and Fe ions’ interactions and the clusters formed inside the amorphous structure. These results demonstrate that manganese ore incorporation significantly influences the structure and the physical properties of calcium aluminosilicate glasses derived from mining tailings, highlighting their potential for technological applications.

## Graphical abstract

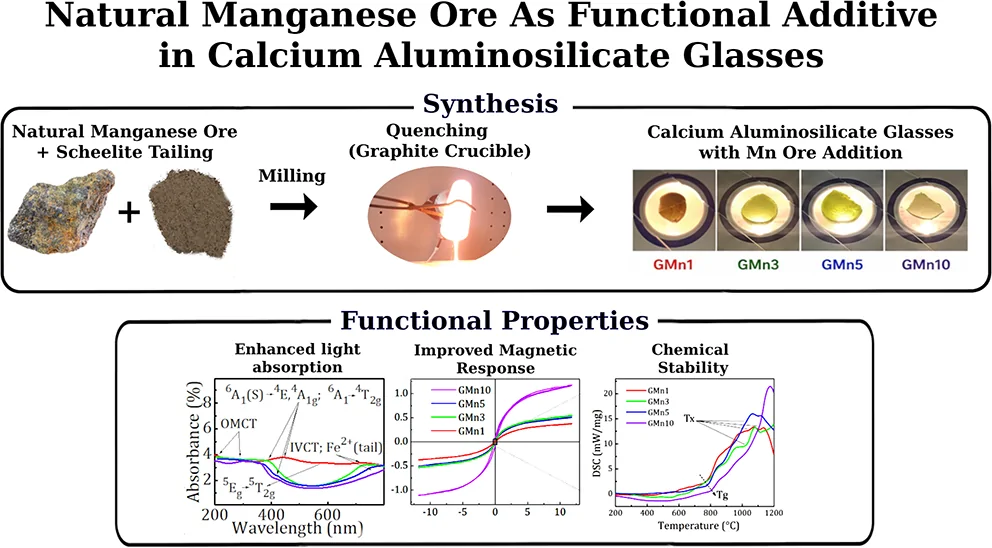

## Introduction

Glasses are materials widely used in many areas due to their versatility and the possibility to manipulate their physical properties. They present an amorphous structure, allowing the incorporation of other elements and materials and making them versatile for technological applications.^1^ For instance, the presence of transition metals changes significantly the optical and magnetic properties of the glasses,^2,3^ and the utilization of mineral wastes as the main precursor for glass synthesis, such as scheelite tailings, is gaining prominence for environmental and economic reasons, which can be enriched with other materials like manganese ore, improving the physical properties and potential applications for the produced glasses.^1,4,5^

The importance of studying the physical properties of glass systems is evidenced in several optical, magnetic, and biomedical applications. Magnetic glasses and glass-ceramics can be utilized in biomedical hyperthermia and bone regeneration, with works highlighting their magnetic properties for targeted therapy and imaging.^6,7^ These materials are also applied in electromagnetic shielding, high-sensitivity magnetic sensors leveraging the GMI effect, environmental remediation as magnetic sorbents, and geoscientific paleointensity studies.^8–12^

Several optical applications of glasses focus on manipulating the valence of transition metals (e.g., Fe and Mn) and incorporating rare earths, resulting in luminescent materials, high-strength windows, and filters.^2,3,9^ These technologies, which include sensors, shielding, and discoloration, are fundamental in the characterization of vitreous and ceramic materials.^13–15^ In iron-containing glasses, iron can be present in different valence states. Fe^2+^ ions produce a broad absorption band in the near-infrared region (≈1000–1100 nm), while Fe^3+^ ions typically absorb in the UV and visible blue regions. The introduction of reducing agents, such as activated carbon, shifts the equilibrium toward Fe^2+^, significantly darkening the glass and altering its spectral transparency.^9^ Glasses doped with rare-earth ions show distinct emission peaks under UV excitation. The intensity and quantum efficiency of this luminescence are highly sensitive to the phonon energy of the glass host.^16^

Some glass systems obtained from industrial waste may precipitate magnetite (Fe_3_O_4_) crystals. These materials show high magnetic softness, making them highly efficient for applications requiring low hysteresis loss.^7^ On the other hand, glass coating introduces significant internal stresses during the fabrication process, which dictate the magnetic domain structure. These microwires are known for extreme magnetic softness and giant magnetoimpedance. Their coercivity is remarkably low, though it can be tuned by removing the glass shell or annealing.^17^

Glasses with manganese and iron obtained from waste may act as ferrimagnets, showing high saturation magnetization, and can be used for high-frequency magnetic radiation absorption, also presenting magnetic coercivity, depending on the size and density of the multidomain magnetite crystals.^4,5^ Studies of transition-metal-doped glasses show that the magnetic ions form clusters within the glass network. These glasses are described as soft-magnetic materials. They exhibit low remanence and coercivity, which is typical for amorphous structures where magnetocrystalline anisotropy is absent or averaged out.^18^ Low coercivity in borosilicate and nickel phosphate glasses is attributed to small magnetic domains and structural disorder in the amorphous matrix, facilitating soft magnetic behavior suitable for sensor applications. Natural volcanic glasses exhibit weak remanent magnetism due to the presence of magnetite nanoparticles.^11,12,19^

Despite these advances, the use of scheelite tailings as a primary precursor for glass production remains relatively unexplored, with only a limited number of studies reported in the literature. Furthermore, the incorporation of natural manganese ore into these systems introduces additional compositional complexity due to the coexistence of multiple oxidation states and mineral phases, which may significantly influence the structural organization of the glass networks. In this context, an aspect that remains insufficiently understood is how manganese addition affects the interplay between structure and multifunctional properties in waste-derived glasses. In particular, the possible formation of metallic or oxide-rich domains at the nanometric scale within the glass matrix, as a function of increasing Mn content, may play a key role in modifying both the magnetic ordering and the optical behavior of the system.^1^ Therefore, investigating these effects is essential to advancing the understanding of disorder-related phenomena in complex glass systems and exploring the potential of sustainable raw materials for technological applications.

In this work, we report the physical properties of calcium aluminosilicate glasses obtained from the scheelite tailings doped with different manganese ore amounts, investigating the structural, calorimetric, optical, and magnetic characteristics of the obtained samples. The selection of the manganese ore is due to the high manganese concentration and the high availability in nature without the need for expensive industrial or chemical processing.

## Experimental Procedure

### Characterization Techniques

The composition of the chemical elements in the synthesized GMnX samples was obtained using a SHIMADZU X-ray fluorescence (XRF) spectrometer, model EDX-7000, equipped with a Rhodium tube with a power of 4 kW. X-ray diffraction (XRD) measurements of the samples were conducted using an X-ray diffractometer (Proto AXRD) with Cu–Kα radiation (λ = 0.154056 nm) in the angular range of 10° ≤ 2θ ≤ 60°, with a step size of 0.0149° and an acquisition time of 5 s, at room temperature. Surface morphological analyses were carried out on powdered glass samples using a high-resolution scanning electron microscope (SEM, Mira3 LMU, TESCAN) equipped with an energy-dispersive X-ray spectroscopy (EDS) detector (Oxford X-ACT). The measurements were performed using secondary electron imaging at an accelerating voltage of 10 kV. Transmission electron microscopy (TEM) was performed using a high-resolution Talos F200i microscope to observe the morphology and internal structure of the glasses in powder form. Molecular vibrational characteristics of the synthesized glasses were obtained via an Agilent Cary 630 spectrophotometer equipped with an ATR-diamond module, with 4 cm^–1^ resolution over a 500–1400 cm^–1^ range in transmittance mode. The glass transition and crystallization temperatures of the samples were determined from thermal property curves obtained using a differential scanning calorimeter model STA 449 Jupiter, coupled with thermogravimetric analysis (DSC-TGA), in a nitrogen atmosphere. The measurements were conducted in the temperature range from room temperature to 1200 °C, with a heating rate of 10 °C/min. Optical absorbance and transmittance spectra were obtained using a VARIAN Cary 50 spectrophotometer, in the wavelength range of 200–800 nm. Electronic paramagnetic resonance (EPR) measurements were obtained from a Bruker CW-EPR EMXmicro spectrometer with a field controller and signal processor. Magnetic hysteresis curves of the glass samples were obtained at room temperature using a vibrating sample magnetometer (VSM, homemade), calibrated using a reference standard (high-purity nickel powder) supplied with the instrument.

### Precursor Composition and Glass Synthesis Procedure

The main precursor is the scheelite tailings doped with different amounts of natural manganese ore, named GMnX, in which X is the wt % of the doped compound. For this study, we used 1, 3, 5, and 10% of natural manganese ore, i.e., GMn1, GMn3, GMn5, and GMn10, respectively. It is relevant to point out that the glass synthesized from the same tailing precursor without Mn incorporation was already studied in previous works.^1,9,10,20^ The main elemental composition of the precursors is detailed in Table 1 below, obtained with X-ray fluorescence (XRF) spectroscopy in powder form and vacuum atmosphere. The crystalline composition of the scheelite tailings is quartz, calcite, dolomite, iron, and other oxides.^1^ The synthesis process was initiated by placing the mixture into a graphite crucible, which was chosen due to its reducing behavior, leading to multivalence states and interesting magnetic and optical behaviors, as shown in previous works,^1,9,20^ and inserting it into the chamber of an electric furnace. The mixture was put in the oven at 1400 °C under air atmosphere, remaining inside for 30 min. Then, the material was removed from the furnace, the molten liquid was poured onto a preheated stainless steel plate at 500 °C and pressed to obtain a smooth surface. Finally, the samples were polished with grits of 300, 1200, 2500, 3000, and 5000 mesh (grain size from 50 to 1.5 μm, respectively).

**Table 1:** Elemental Composition of Scheelite Tailings and Manganese Ore Used to Synthesize the GMnX Samples Obtained from XRF Measurements

Element	Tailings	Manganese Ore
Ca	53.1%	1.0%
Si	30.0%	4.3%
Al	10.7%	3.8%
Fe	3.1%	14.7%
K	1.3%	1.5%
Mn	0.4%	73.9%
Others	1.4%	0.8%

## Results and Discussion

### Structural Characteristics

#### Elemental Composition of the Glasses

The elemental compositions of the synthesized GMnX glasses in powder form, obtained by X-ray fluorescence (XRF) under vacuum atmosphere, are shown in Table 2. Calcium and aluminum presented a relative decrease with Mn ore doping, as well as S, K, and other elements. Iron presented small relative amount variations among the samples, and Mn increased almost linearly with manganese ore doping increase. The nonlinear variations of some elements are attributed to the use of tailings and ore, which may present a not uniform elemental distribution. Especially regarding Fe, since both precursors present iron in significant amounts (although different), by replacing part of scheelite tailings with Mn ore, no great changes are expected to be observed. In addition, the use of natural and waste precursors may not provide an equal elemental distribution among the samples. These factors may have led to the observed results relative to the Fe amounts.

**Table 2:** Elemental Composition of the Synthesized GMnX Samples Obtained from XRF Measurements^a^

	Element amount (%)							
Sample	Ca	Si	Mn	Al	Fe	S	K	Others
GMn1	53.3	26.4	2.4	5.4	5.6	2.8	2.5	1.6
GMn3	56.1	22.4	5.7	5.3	5.4	2.3	1.5	1.3
GMn5	55.7	22.7	8.2	4.8	3.6	2.2	1.8	1.0
GMn10	49.2	21.8	14.6	5.2	4.6	2.0	1.6	1.0

a Manganese content increases with the ore addition.

#### XRD Results

The X-ray diffraction (XRD) patterns shown on the left side of Figure 1 correspond to the GMnX samples (X = 1, 3, 5, and 10 related to the Mn ore incorporation percentage). The GMn1 sample exhibits a broad diffuse scattering centered in the 20–35° (2θ) range, which is characteristic of a typical amorphous glassy structure commonly observed in silicate glasses.^10,20^ The absence of sharp diffraction peaks confirms the lack of long-range crystalline order. Although the amorphous pattern remains dominant, slight changes in its shape and intensity suggest structural rearrangements within the glass network with Mn addition. Other structural features are discussed in the next results.

**Figure 1:**
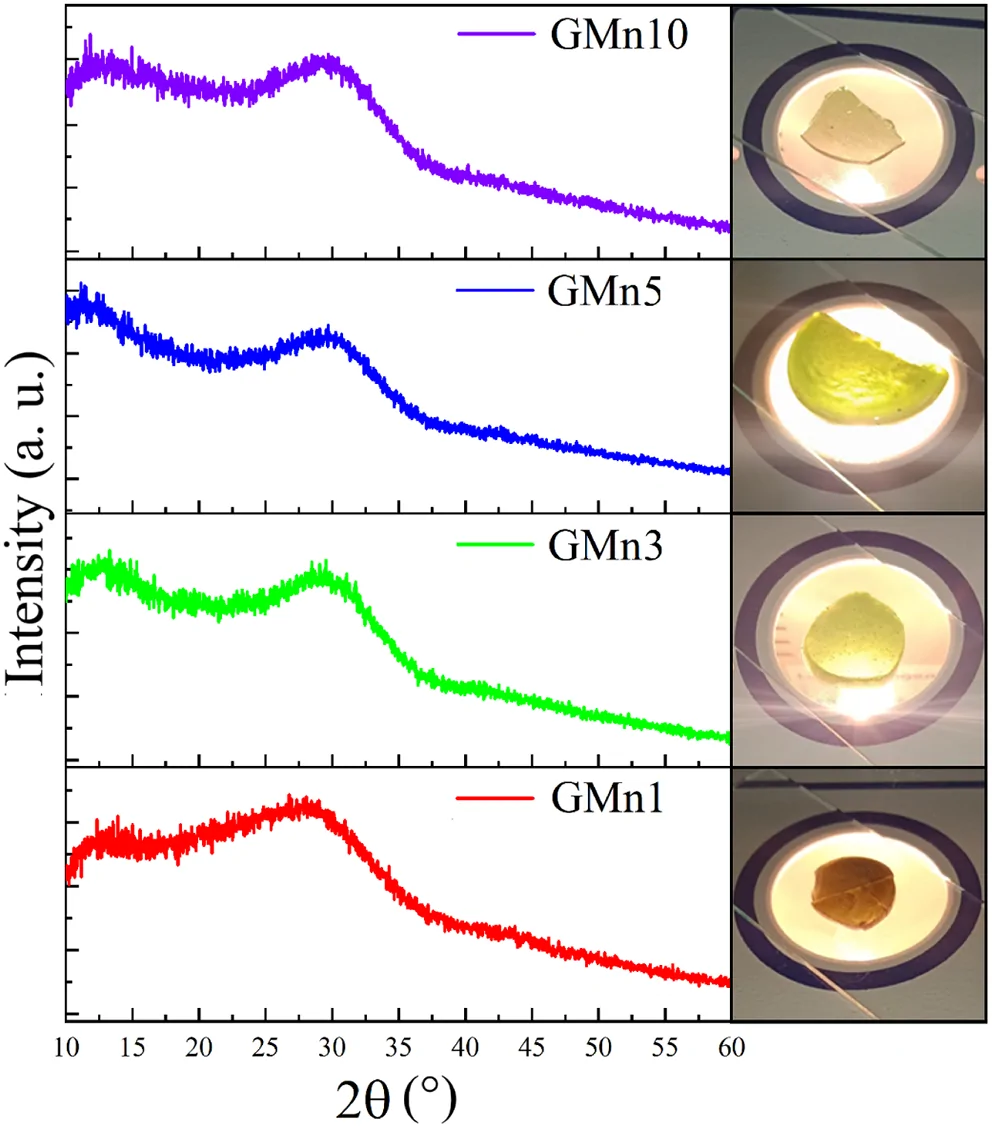
X-ray diffraction (XRD) patterns of the GMnX glass samples (X = 1, 3, 5, and 10 wt % Mn ore) recorded in the 10–60° (2θ) range (left). The photographs on the right show the corresponding synthesized glass samples, highlighting the variation in color with Mn content variation.

At the right side of Figure 1, the macroscopic appearance of the GMnX samples is presented, showing that all compositions exhibit a greenish coloration attributed to iron ion reduction, as will be detailed in the following results.^10,20–22^ Interestingly, as the Mn concentration increases, the transparency of the samples becomes more pronounced, suggesting that manganese influences the redox equilibrium and optical absorption properties of the material,^23,24^ which is detailed in the optical results. Additionally, all samples display noticeable porosity and microspherical features observed by optical microscopy, indicating incomplete homogenization during melting, possibly due to increased melt viscosity associated with higher manganese content. Moreover, the Mn percentage increase leads to a higher melting temperature, which may further hinder full densification and contribute to the observed porosity.

#### SEM/EDS Analysis

Figure 2 shows, on the left, scanning electron microscopy (SEM) images of the glass in powder form and, on the right, the respective elemental distribution maps obtained by energy-dispersive X-ray spectroscopy (EDS), with a scale of 250 μm. The SEM micrographs reveal glassy fragments of irregular morphology and different sizes, characteristic of the maceration process used to obtain the powders. The EDS maps show a relatively homogeneous distribution of the elements, indicating good chemical homogeneity. Additionally, Table 3 presents the elemental composition of the samples obtained by EDS. A gradual increase in manganese concentration is also observed, from 1.0 at. % in sample GMn1 to 6.0 at. % in sample GMn10, accompanying the increase in the amount of manganese ore. Iron exhibits concentrations ranging between 1.5 and 3.1 at. %. Although both XRF and EDS results corroborate, the differences observed are associated with the distinct operating principles, in which XRF provides the average chemical composition of the bulk material, and SEM/EDS performs a localized analysis volume near the sample surface. Furthermore, Fe- and Mn-containing particles are dispersed throughout the glass matrix, in accordance with the following results.

**Table 3:** Elemental Composition of the GMnX Samples in Powder Form Obtained from EDS Measurements, Showing the Mn Content Increase with Ore Addition

	Element amount (%)								
Sample	O	Ca	Si	Mn	Al	Fe	K	Mg	Na
GMn1	53.0	18.1	17.8	1.0	4.4	2.4	0.9	1.8	0.7
GMn3	60.2	16.2	13.7	1.6	3.8	1.7	0.6	1.6	0.5
GMn5	56.5	19.5	13.7	2.7	3.4	1.5	0.7	1.4	0.6
GMn10	50.2	19.4	14.5	6.0	4.0	3.1	0.5	1.6	0.6

**Figure 2:**
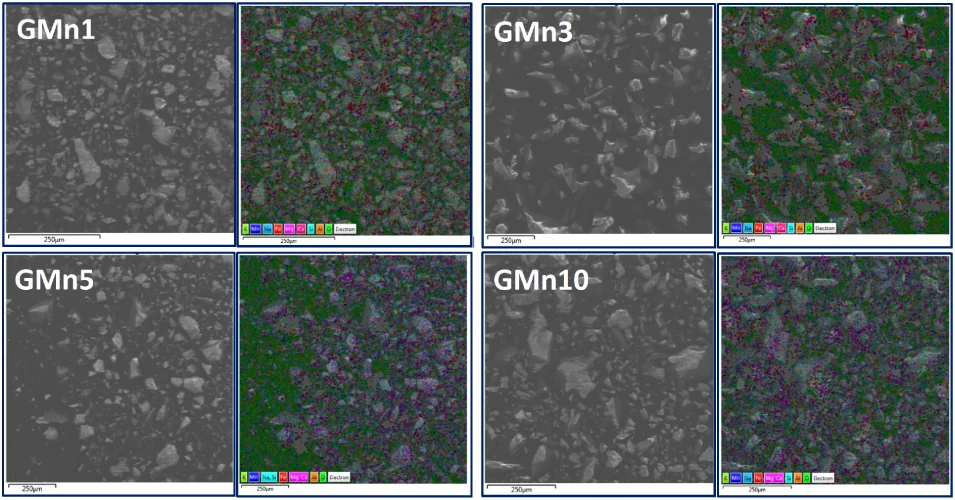
SEM/EDS images for the GMnX glasses in a 250 μm scale, evidencing the approximately homogeneous elemental distribution in the glasses.

#### TEM Measurements

Figure 3 shows the results for the Transmission Electron Microscopy (TEM) measurements for the GMn5 and GMn10 glasses. On the left (Figure 3a) we have the TEM images at a 20 nm scale for GMn5 and GMn10, respectively, in which we notice an approximately uniform background containing small dark spots, attributed to nanodomains or ion clusters formed during the synthesis process in the glass matrix due to manganese ore doping.^25^ In GMn5 (above), the particles are dispersed in the glass matrix, while in GMn10 (below), we can notice a more intense clustering of particles due to the greater amount of manganese ore doping, resulting in a more intense magnetic behavior, as discussed later. Although the TEM results indicate the presence of nanodomains for these samples, XRD did not present crystalline peaks. In Figure 3b, we have the TEM image for GMn10 at a 10 nm scale, in which we observe nanodomains or ion clusters formation in the glass matrix. Complementarily, Figure 3c shows the estimated particle size distribution for the GMn5 and GMn10 samples, varying between 1 and 5 nm for both samples, in which GMn5 presented an average size of 2.5 nm and GMn10, 3.5 nm. The distribution was analyzed over 50–60 particles approximately. Due to the not-well-defined particle edges, the size estimation may vary around ±0.2 nm. Besides, the 2D visualization may be showing particles in different depths as a surface, hindering the particles’ distinction. The size distribution difference is attributed to the greater manganese ore amount in GMn10 in comparison to GMn5.

**Figure 3:**
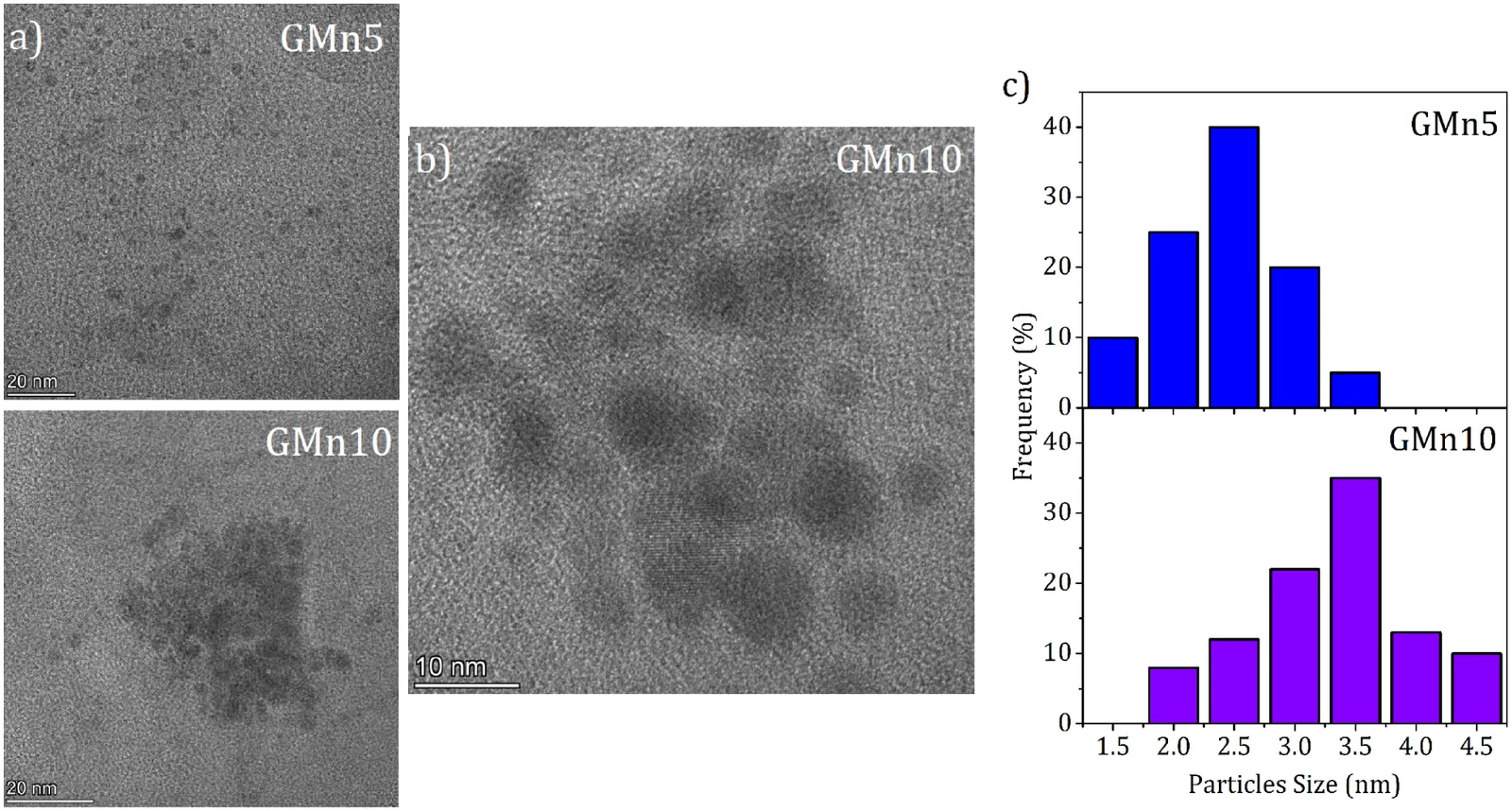
Transmission electron microscopy results for the GMn5 and GMn10 glass samples at a) 20 nm and b) 10 nm scales. The spots indicate clusters/nanoparticles formation inside the glass matrix due to the manganese ore addition, detailed in the c) size distribution histogram for GMn5 (top) and GMn10 (bottom) samples over 50–60 particles with ±0.2 nm variation, showing greater average size for the GMn10 sample.

#### FTIR Structural Analysis

The Fourier transform infrared (FTIR) spectra of GMnX calcium aluminosilicate glasses, shown in Figure 4, exhibit the typical vibrational characteristics of silicate-based glass lattices [1,2]. In general, the spectra can be divided into two main spectral regions: (i) the intermediate region, between 600 and 800 cm^–1^ (light blue), and (ii) the high-frequency region, above 800 cm^–1^ (light purple). It should be noted that the measurements were performed from 500 cm^–1^, making it impossible to obtain detailed information in the low-frequency region (<600 cm^–1^). The high-frequency region (800–1200 cm^–1^) is dominated by an intense and broad absorption band centered approximately between 950 and 1100 cm^–1^. This band is attributed to the asymmetric stretching vibrations of the Si–O–Si and Si–O–Al bonds, corresponding to the bridging oxygen (BO) atoms that connect the [SiO_4_] and [AlO_4_] tetrahedra.^26,27^ These tetrahedral units constitute the main structure of the silicate glass network. Furthermore, with increasing Mn ore content from GMn1 to GMn10, a shift in this band is observed.

**Figure 4:**
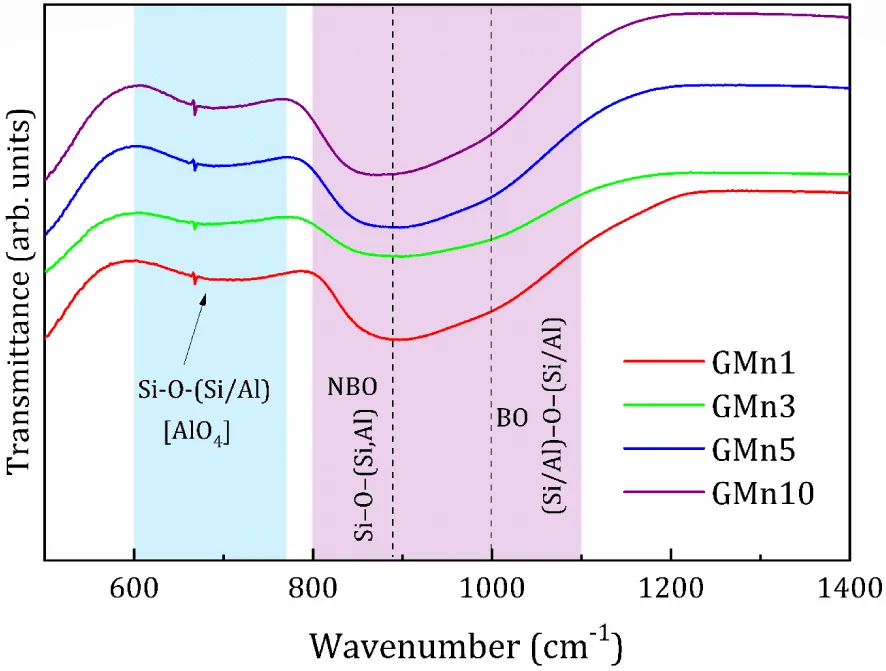
FTIR transmittance spectra of GMnX calcium aluminosilicate glasses (X = 1, 3, 5, and 10 wt % Mn ore) recorded in the range of 500–1400 cm^–1^.

In GMn1, the BO band remains relatively well-defined, indicating a more polymerized structure. However, as the Mn concentration increases, the main BO band shifts slightly to lower wavenumbers and becomes wider. This behavior indicates a reduction in the network connectivity.^23^ Simultaneously, a band or shoulder around ≈890 cm^–1^ becomes more evident with increasing Mn content. This band is attributed to the stretching vibrations of nonbonding oxygen (NBO) bonds, i.e., Si–O^–^ groups formed due to the breaking of Si–O^–^–[Si,Al] bridges. The intensity increase of this NBO-related feature suggests that manganese incorporation promotes structural rearrangements and the formation of nonbridging oxygen environments. Considering the multiphase nature of the manganese ore precursor, manganese may occupy different environments in the glass matrix. Therefore, the observed spectral evolution is consistent with a modifier-like behavior, although contributions from intermediate structural roles cannot be excluded.^26,27^ By introducing additional cations into the glassy matrix, charge compensation and structural rearrangements occur, promoting the formation of NBO sites and, consequently, the depolymerization of the silicate network.^28,29^ In the intermediate region (600–800 cm^–1^), bands associated with Si–O^–^[Si/Al] bending modes and possible contributions of [AlO_4_] units are observed.^26,27^

### Thermal Studies

#### Calorimetric Transitions and Glass Stability

The calorimetric analysis for the GMnX samples is shown in Figure 5, presenting the phase transitions of the studied glasses using differential scanning calorimetry (DSC), from which we can estimate the main glass transitions in function of the temperature and other important parameters, such as the thermal stability of the glass. *T*_*g*_ is indicated by an endothermic event that confirms the amorphous phase formation of the glass matrix. For the GMnX samples, the estimated values were between 781 and 809 °C. *T*_*x*_ is an exothermic event that represents the onset of glass crystallization, resulting in values between 1032 and 1116 °C for the studied glasses. Figure 5a shows the DSC measurements overlap for the GMnX samples, and Figure 5b indicates the estimates of *T*_*g*_ and *T*_*x*_, based on the exothermic and endothermic events of the curve. Table 4 summarizes the obtained values for the transition parameters.

**Figure 5:**
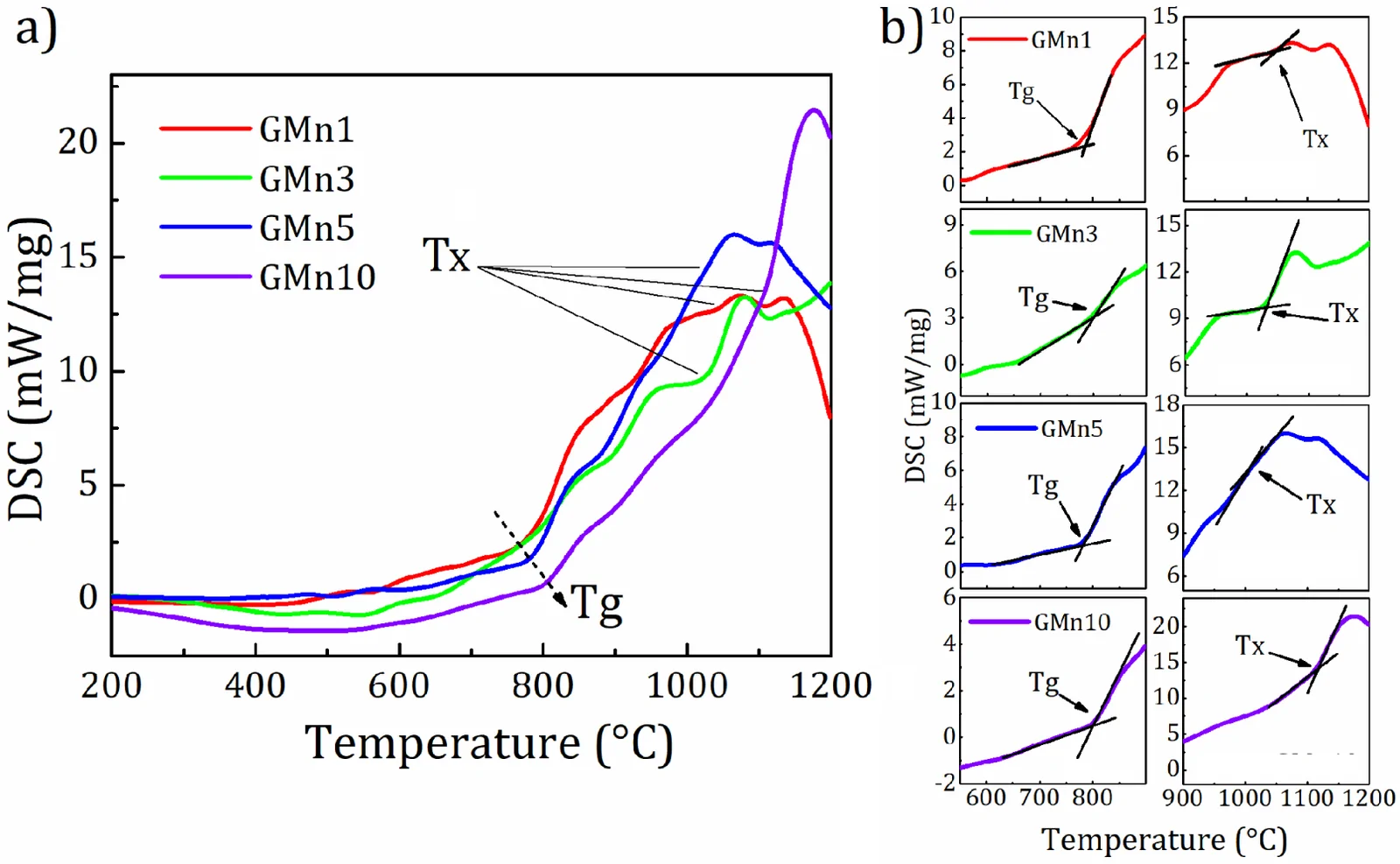
(a) DSC curves of the GMnX samples (X = 1, 3, 5, and 10 wt % Mn ore) recorded from 200 to 1200 °C, showing the glass transition temperature (*T_g_*) and the onset of crystallization temperature (*T_x_*). (b) Enlarged DSC curves highlighting the determination of *T_g_* (baseline shift) and *T_x_* for each composition.

**Table 4:** Glass Transition (*T_g_*), Onset of Crystallization (*T_x_*) Temperatures, and the Thermal Stability *H_R_* Parameters for the GMnX Samples

Sample	*T*_*g*_ (°C)	*T*_*x*_ (°C)	Δ*T* = *T*_*x*_ – *T*_*g*_ (°C)	*H*_*R*_ factor
GMn1	781.0 ± 1.0	1039.0 ± 1.0	258 ± 2.0	0.60
GMn3	787.0 ± 1.0	1032.0 ± 1.0	245 ± 2.0	0.57
GMn5	785.0 ± 1.0	1033.0 ± 1.0	248 ± 2.0	0.57
GMn10	809.0 ± 1.0	1116.0 ± 1.0	307 ± 2.0	0.95

To quantify the thermal stability of the GMnX samples, we used the Hruby factor (*H*_*R*_) calculated using the melting point of cordierite (*T*_*m*_ = 1467 °C) due to its compositional similarity to the studied aluminosilicate glass samples, in accordance with similar works,^1,28,30,31^ as well as the estimated *T*_*g*_ and *T*_*x*_, defined as:

$$H_{R}=\frac{T_{x}-T_{g}}{T_{m}-T_{x}}$$

The results, also shown in Table 4, remained around 0.60 for GMn1, GMn3, and GMn5 samples and resulted in 0.95 for GMn10, indicating a good thermal stability, which can be due to the presence of Fe^2+^ acting as a network modifier, enhancing the thermal stability of the glass. Manganese incorporation does not affect thermal stability up to 5% Mn ore addition but is slightly improved for the GMn10 sample. Although manganese incorporation may promote local structural modifications within the glass network, it may also increase the thermal stability by raising *T*_*g*_ and *T*_*x*_. This happens because manganese may form stronger atomic bonds and delay crystallization,^23,32^ which is most prominent in the GMn10 sample.^33^

### Optical Studies

#### UV–Visible Electronic Transitions

The spectroscopic analysis of transition metal ions such as iron and manganese in glass matrices is fundamental to understanding the local structure and the nature of chemical bonding within the material. In addition, determining the Racah parameters B and C from ultraviolet–visible spectroscopy (UV–vis) measurements allows the quantification of the d-electron cloud due to the covalency of the metal–oxygen bond.^32^ According to the literature, Fe^2+^ absorbance exhibits a broad, intense absorption band in the near-infrared (NIR) region, typically centered around 1000–1100 nm. This band corresponds to the ligand field transition in octahedral symmetry.^34^ Fe^3+^ displays several weak Laporte-forbidden transitions around 380, 420, and 440 nm, as well as a strong charge transfer band in the UV region, which causes the yellow-brown tint in glass.^15,35^ Mn^2+^ often exhibits a pale pink or nearly colorless glass due to ^6^ *A*_1_ → ^4^ *T*_2*g*_ spin-forbidden transitions from a d^5^ configuration around 430 nm,^36,37^ and Mn^3+^ shows a strong absorption in the visible region, with a peak around 500 nm due to an ^5^ *E*_*g*_ → ^5^ *T*_2*g*_ transition, producing an intense purple/violet color.^38^ The interaction between Fe and Mn acts as a key chemical buffer, with charge transfers between Fe^2+^, Fe^3+^, Mn^2+^, and Mn^3+^ ions, in which Mn neutralizes the green tint provided by Fe. The reaction may shift the iron to weak yellow and manganese to nearly colorless, resulting in a visually clear glass.^35^

Figure 6 shows the UV–vis spectroscopy results for the GMnX samples, with transmittance and absorbance results and the band gap estimate. Transmittance plots show a transmission increase with manganese ore addition increase, confirmed by the visual characteristics of the GMnX samples in Figure 1, attributed to the combined Mn and Fe ion transitions, as discussed above. The absorption results are shown in the inset. The absorption signal in the UV region (around 300 nm) is an oxygen-to-metal charge transfer (OMCT), in which an electron is promoted from the 2*p* orbital of O^2–^ to the 3*d* orbital of the Fe^3+^.^39^ The peaks at 385 nm (GMn3), 420 nm (GMn5 and GMn10), and 440 nm (GMn1) correspond to the transition from the Fe^3+^ ground state (^6^ *A*_1_) to the doubly degenerate ^4^ *E* and ^4^ *A*_1_ excited states. In a high-spin configuration, this transition requires a change in spin, making these transitions spin-forbidden and therefore weak, indicating that Fe^3+^ ions enter tetrahedral sites and act as network formers.^39–42^ This region is also related to ^5^ *E*_*g*_ → ^5^ *T*_2*g*_ transitions from Mn^3+^ for GMn1, and ^6^ *A*_1*g*_ → ^4^ *T*_2*g*_ from Mn^2+^ for all samples, as discussed before. According to literature, the broad absorption appearing around 700–800 nm extending into the near-infrared is typically associated with IVCT (inter-valence charge transfer) between Fe^2+^ and Fe^3+^ ions, as well as with Fe^2+^ d–d transition usually centered around 1000–1100 nm, but its tail begins near 700 nm, giving the glass a characteristic green or bluish tint.^34,43^ Table 5 shows the main transitions identified for the GMnX samples.

**Figure 6:**
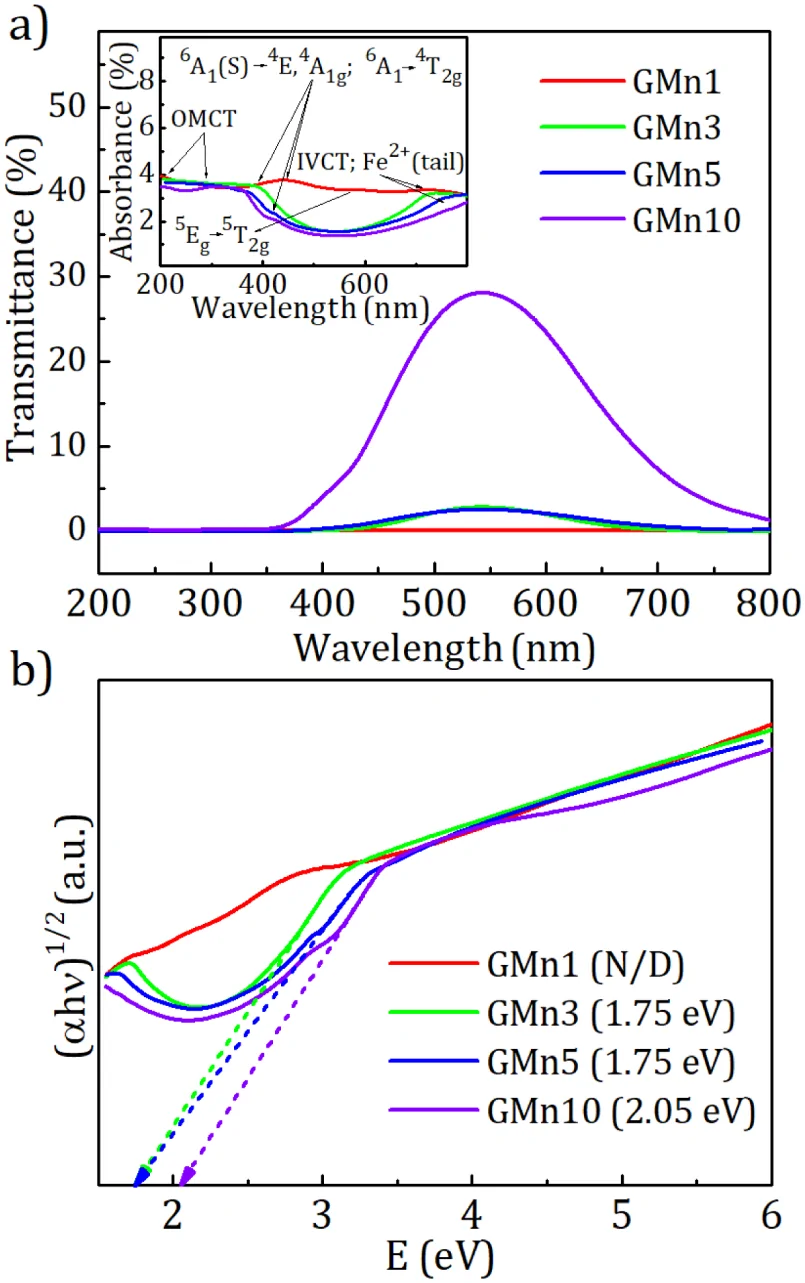
UV–visible spectroscopy results for the GMnX glasses with a) transmission and absorption with electronic transitions at the inset and b) estimated band gap, remaining between 1.75 and 2.05 eV for the samples, except for GMn1 (not determined).

**Table 5:** Electronic Transitions and Spectral Assignments for the Calcium Aluminosilicate Glass Samples

Wavelength (nm)	Electronic Transition	Type
∼250–350	O^2–^ → Fe^3+^	OMCT
∼380–440	^6^ *A*_1_ → ^4^ *E*, ^4^ *A*_1_(*G*)	d–d
	^6^ *A*_1_ → ^4^ *T*_2*g*_	
∼500–600	^5^ *E*_*g*_ → ^5^ *T*_2*g*_	d–d
∼700–800	Fe^2+^ → Fe^3+^	IVCT

#### Racah Parameters

To perform the Racah parameters calculation, the field-independent transition for high-spin d^5^ systems is identified in the absorption spectrum, corresponding to the level ^6^ *A*_1_(*S*) → ^4^ *E*, ^4^ *A*_1*g*_. For GMn5 and GMn10, the sharp peak is located at 420 nm (23,809 cm^–1^). According to the Tanabe–Sugano ligand field theory, the energy of this specific transition is given by the equation:

E=10B+5C

in which, for oxide glasses, we use the approximation *C* = 4*B*, resulting in *E* = 30*B*.^44,45^ Therefore, we obtain *B* = 793 cm^–1^ and *C* = 3,172 cm^–1^. Additionally, the Ion Racah Parameter represents the interelectronic repulsion in a metal ion that is completely isolated in a vacuum, with no surrounding ligands.^41^ From the standardized value for Fe^3+^ (1015 cm^–1^), we can estimate the nephelauxetic ratio (β), which represents the cloud-expanding effect in transition metal complexes and is defined as:

$$\beta =\frac{B_{glass}}{B_{free‐ion}}$$

In this case, *B*_*glass*_ = 793 cm^–1^, resulting in β = 0.78 for the GMn5 and GMn10 samples, indicating that the Fe^3+^ bonds in the calcium aluminosilicate matrix have a significant covalent (less ionic) character, reinforcing its network former properties.^46^ Table 6 summarizes the estimated parameters for the studied glasses.

**Table 6:** Calculated Racah Parameters and Nephelauxetic Ratio for the GMn5 and GMn10 Samples

Parameter	Calculated Value	Observation
Peak Energy (*E*)	23,809 cm^–1^	Transition energy for the ^4^ *E*, ^4^ *A*_1_ state
Racah Parameter *B*	793 cm^–1^	Interelectronic repulsion in the glass matrix
Racah Parameter *C*	3,172 cm^–1^	Assuming *C* ≈ 4*B*
Nephelauxetic Ratio (β)	0.78	∼22% covalency in the Fe–O bond

#### Band Gap Estimative

Finally, we estimate the band gap using Tauc’s plot model from the absorbance coefficient (Figure 6b). This model is based on near band edge energy photon absorption, following the equation

$${\left(\alpha h\nu \right)}^{1/n}=A\left(h\nu -E_{g}\right)$$

in which α is the absorption coefficient obtained from the absorption measurement, *h*ν is the incident photon energy, *n* defines the electron transition (*n* = 2 is the standard value for most glass systems), and *E*_*g*_ is the band gap energy. The band gap is estimated by tracing the plot’s linear part to the energy axis,^1,9,47,48^ resulting in values between 1.75 (±0.10) and 2.05 (±0.10) eV for the GMnx samples, in accordance with similar glass systems with iron and manganese, except for GMn1, for which we could not determine the band gap possibly due to impurities or defects in the sample, which may have influenced the absorbance measurement.^9,39,49,50^

Determining these parameters has direct implications for the glass’s technological applications. From an optical standpoint, a reduction in *B* correlates with an increase in the intensity of absorption bands and a shift in the ultraviolet absorption edge (OMCT), which is essential for UV-protective glasses.^51^ Structurally, the high covalency suggested by the low *B* value indicates that iron is strongly bonded to the glass network. This contributes to enhanced chemical durability and resistance to hydrolytic attack, as the ion ceases to act as a mobile network modifier and becomes a stable structural component.^39^ Therefore, controlling Racah parameters through chemical composition allows for the engineering of glasses with customized optical and mechanical properties for industries requiring high stability.

### Magnetic Properties Studies

#### EPR Analysis

Figure 7a–d presents the EPR spectra of the GMnX samples (X= 1, 3, 5, and 10%) glasses measured at room temperature. All spectra exhibit a broad resonance signal centered in the magnetic field region corresponding to *g* ≈ 2.00–2.07, which is characteristic of transition-metal ions with a 3d^5^ electronic configuration, such as Mn^2+^ and Fe^3+^ (S = 5/2) in oxide glass matrices.^20,52^ In amorphous systems, without long-range structural order, transition-metal ions are typically distributed randomly within the glass network, leading to broadened EPR signals associated with isolated paramagnetic centers.^53^ The experimental spectra were fitted using a derivative line-shape model corresponding to the first derivative of the absorbed microwave power with respect to the magnetic field. The fitting expression can be written as:

$$\frac{dP}{dH}=A_{sym}\frac{4\Delta H_{pp}\left(H-H_{r}\right)}{{[4{\left(H-H_{r}\right)}^{2}+{\left(\Delta H_{pp}\right)}^{2}]}^{2}}-A_{asym}\frac{{\left(\Delta H_{pp}\right)}^{2}-4{\left(H-H_{r}\right)}^{2}}{{[4{\left(H-H_{r}\right)}^{2}+{\left(\Delta H_{pp}\right)}^{2}]}^{2}}+C$$

In this expression, *H*_*r*_ denotes the resonance magnetic field, Δ*H*_*pp*_ the peak-to-peak line width, and *C* the baseline offset. However, the spectroscopic *g*-factor was determined using the resonance condition

$$g=\frac{h\nu }{\mu_{B}H_{r}}$$

in which *h* is Planck’s constant and μ_*B*_ is the Bohr magneton.^54,55^

**Figure 7:**
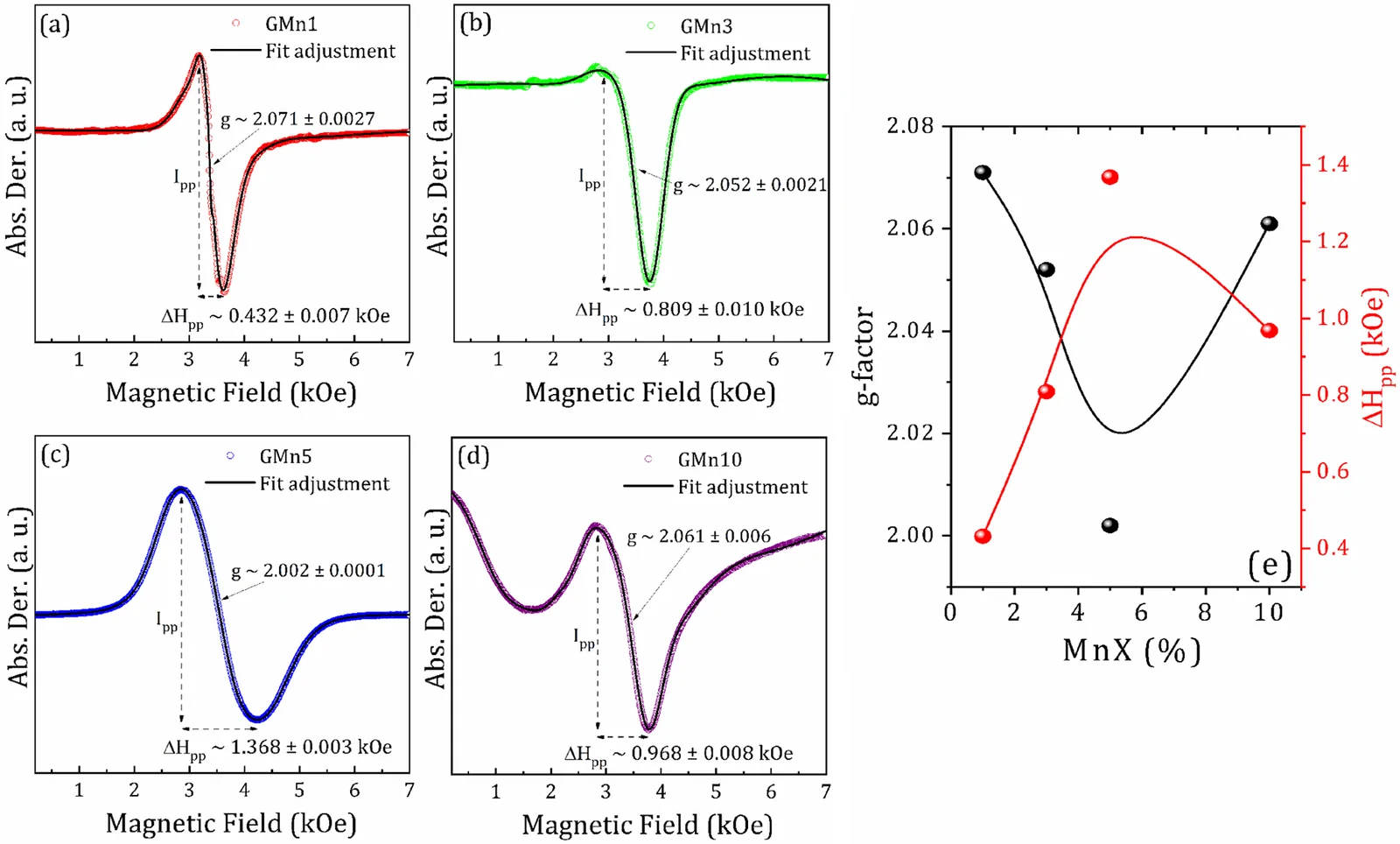
EPR spectra of the GMnX glass samples (X = 1, 3, 5, and 10 wt % Mn ore) measured at room temperature. Panels (a)–(d) show the experimental spectra and their corresponding fits using a derivative line-shape model (first derivative of the absorbed microwave power) for GMn1, GMn3, GMn5, and GMn10 samples, respectively. (e) The variation of the *g*-factor and the peak-to-peak line width (Δ*H_pp_*) as a function of Mn content.

For the samples with lower manganese content (GMn1 and GMn3), the resonance lines are relatively narrower, suggesting that Mn^2+^ and Fe^3+^ ions are mostly isolated within the glass network.^56^ However, a clear line broadening is observed for the GMn5 and GMn10 samples, as evidenced by the increase in the peak-to-peak line width (Δ*H*_*pp*_) shown in Figure 7e. The first hypothesis for the observed line width broadening is related to the strengthening of magnetic interactions between transition-metal ions as the manganese concentration increases. The progressive increase in line width suggests enhanced dipole–dipole interactions among Mn^2+^ ions, as well as superexchange interactions between Mn^2+^ and Fe^3+^ ions mediated by oxygen bridges within the glass network (Fe–O–Mn).^20^ As the Mn content increases, the average distance between paramagnetic ions decreases, which intensifies these magnetic interactions and consequently leads to line broadening in the EPR spectra. The second hypothesis is associated with the formation of nanometric magnetic clusters within the glass matrix. The significant line width broadening observed in the GMn5 and GMn10 samples may be related to this effect.^38,57^

#### VSM Results and Discussion

Figure 8 shows the magnetic measurements results for the GMnX glasses. Figure 8a shows the hysteresis curves (magnetization versus applied field) for the samples, obtained from a vibrating sample magnetometer (VSM). We can notice an overlap of magnetic behaviors in all samples, with saturation magnetization, magnetic susceptibility, and a small coercivity. The magnetization increases with mineral manganese doping increase, varying approximately from 0.38 emu/g in GMn1 to 1.20 emu/g in GMn10 for a maximum applied field of 12 kOe (GMn3 and GMn5 present a small inversion for this trend, but their magnetization values are very close to each other). For comparison, studies on similar glasses showed *M*_*S*_ between 0.1 and 0.5 emu/g, and χ between 0.8 and 20 emu/g·Oe, approximately.^9,16^ These results indicate a consistent magnetic behavior for these glasses, with a greater variation only for the GMn10 sample due to the Mn addition amount. Figure 8b highlights the central region of the curves to evidence the coercivity (*H*_*C*_), also presenting an increase with Mn mineral doping increase, resulting in values between 85 and 150 Oe. Figure 8c presents the Langevin plus linear fits for the magnetization measurements, i.e.,

$$M=M_{S}\left[\coth\left(a\right)-1/a\right]+\chi H$$

in which *a* is an applied field (*H*) function and is also related to the magnetic moment μ, as will be discussed later. Although the samples present coercivity, this model works with great approximation in this case due to the very low *H*_*C*_ values of the samples. The Langevin fit estimates the saturation magnetization (*M*_*S*_) related to the frustrated ferromagnetic interactions, and the linear fit estimates the magnetic susceptibility (χ) related to paramagnetic and frustrated antiferromagnetic interactions. Both *M*_*S*_ and χ show an increasing trend with mineral Mn doping increase, as shown in Figure 8d, with GMn10 presenting the most *M*_*S*_ absolute and relative increase among the samples due to the greater manganese ore doping amount. Table 7 summarizes the obtained results. The discussion of these behaviors is as follows.

**Figure 8:**
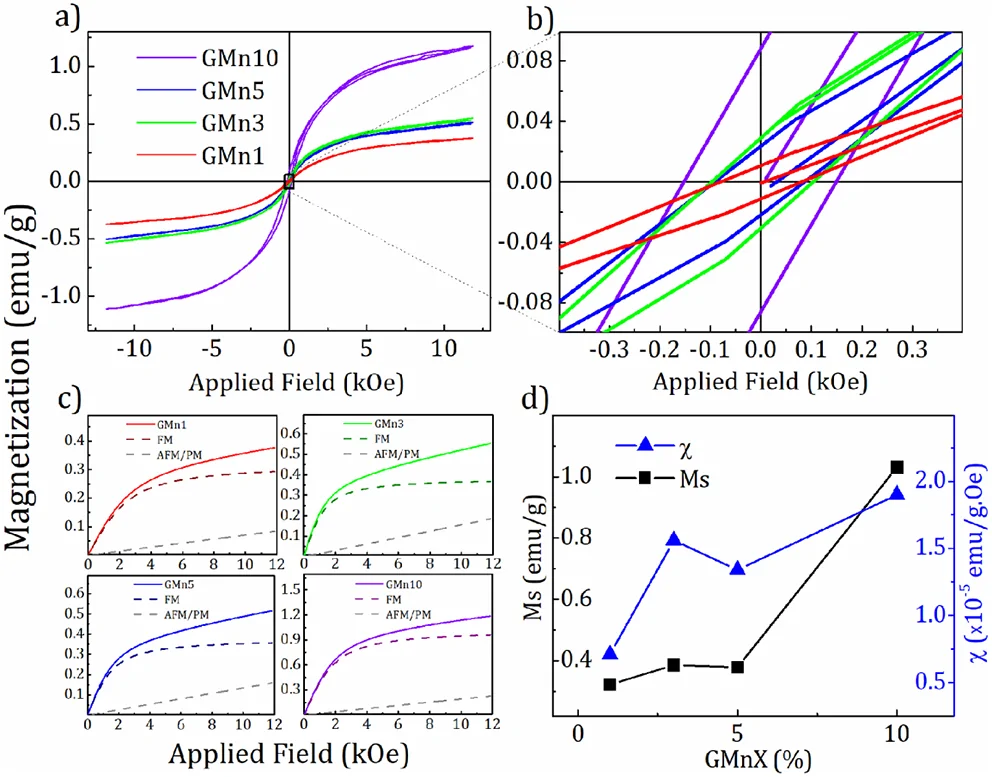
Magnetization measurements taken at room temperature (300 K) for the GMnX glasses: a) overlapped, b) amplified for coercivity visualization, c) magnetization fits, and d) *M_S_* and χ behaviors.

**Table 7:** Magnetic Parameters Obtained from Langevin Plus Linear Fits for the GMnX Glasses

Sample	Coercivity (Oe)	Remanence (emu/g)	*M*_*S*_ (emu/g)	χ (emu/g·Oe)
GMn1	82	0.011	0.322 ± 0.006	(0.7 ± 0.05) × 10^–5^
GMn3	103	0.029	0.386 ± 0.012	(1.5 ± 0.11) × 10^–5^
GMn5	89	0.024	0.379 ± 0.008	(1.3 ± 0.07) × 10^–5^
GMn10	150	0.088	1.031 ± 0.032	(1.9 ± 0.27) × 10^–5^

Typically, in glasses with low iron and manganese concentrations, transition-metal ions such as Fe^2+^, Fe^3+^, and Mn^2+^ can be distributed randomly in the network, leading to paramagnetic behavior. In addition, manganese-containing glasses often exhibit antiferromagnetic interactions between Mn ions at low temperatures, transitioning to paramagnetism at room temperature.^58^ In complex silicate glasses containing amounts of iron, unique, disordered magnetic structures may arise, known as asperomagnetism (ASPM) and speromagnetism (SPM). These behaviors arise from frustrated antiferromagnetic and ferromagnetic ion interactions caused by the disordered glass matrix, possibly presenting weak ferromagnetism when short-range magnetic order is present or spin glass. When the ion interactions are predominantly ferromagnetic, provided from Fe^2+^ and Fe^3+^ superexchange coupling, we have ASPM, and when they are majorly antiferromagnetic from ions with the same valence state, we have SPM. The addition of activated carbon or the utilization of graphite crucibles acts as a reducing agent, converting Fe^3+^ to Fe^2+^, facilitating superexchange interactions within the amorphous matrix.^1,9,20,59^ Additionally, the presence of ion clusters and nanoparticles may provide behaviors such as superparamagnetism and cluster glass.

In higher concentrations of iron and manganese oxides, magnetic nanoparticles may form, such as ferrites.^60,61^ Iron- and manganese-containing glasses derived from industrial wastes may lead to significant crystallization during or after synthesis, possibly forming magnetite, maghemite, or jacobsite.^62,63^ Controlled crystallization of some glasses may lead to the formation of ferrite nanostructures, exhibiting a ferromagnetic/ferrimagnetic state, with low saturation magnetization and coercive force.^64^

For the GMnX glasses, the utilization of scheelite tailings, manganese ore, and graphite crucible resulted in the combination of the behaviors discussed above, attributed to the formation of Fe and Mn ion clusters with complex magnetic interactions. In this case, as already studied in previous works for glasses synthesized from the same tailing precursor,^1,9,10,20^ we attribute the main magnetic behavior to frustrated ferromagnetic and antiferromagnetic ion interactions within the amorphous glass structure, i.e., ASPM, resulting in the observed *M*_*S*_ and χ from the measurements, especially for the least Mn-incorporated samples, GMn1 and GMn3. Furthermore, cluster formations were observed in the TEM images, mainly in GMn10, improving its magnetization. Due to the presence of nanodomains and ion clusters, a significant superparamagnetic-like contribution, such as cluster glass, may also occur. The combination of these behaviors is assumed to be present in the samples in different degrees according to the manganese addition, providing the observed *H*_*C*_, *M*_*S*_, and χ values obtained from the fit.

#### Magnetic Moment

We can also estimate the magnetic moment (μ) from the Langevin function’s results using the fit values obtained for GMn10, and

$$a=\mu H/k_{B}T$$

in which *k*_B_ is the Boltzmann constant (1.38 × 10^–16^ erg/K) and *T* = 300 K, with *a* = 1.03 provided by the fit (for *H* in kOe), resulting in μ = 5.13 × 10^–17^ erg/Oe (≈5.54 μ_*B*_). This result indicates that most of the ions are majorly scattered in the glass matrix, providing the ASPM behaviors from the frustrated ferromagnetic and antiferromagnetic ion interactions and cluster glass due to the presence of clusters of iron and manganese in the glass matrix.^65–68^

## Conclusions

In this work, calcium aluminosilicate glasses derived from scheelite tailings doped with different concentrations of natural manganese ore were successfully produced by using the conventional melt-quenching method. Structural results confirmed the formation of a predominantly amorphous matrix, indicating the effective incorporation of Mn into the system. Spectroscopic analyses indicated that manganese addition promotes structural modifications in the silicate network, including an increase in nonbridging oxygen sites and partial depolymerization of the glass network, consistent with modifier-like behavior. Transmission electron microscopy (TEM) observations, performed for the GMn5 and GMn10 samples, revealed the presence of nanodomains dispersed within the glass matrix with a greater tendency toward clustering at higher manganese concentrations. Thermal analyses demonstrated good thermal stability of the materials with well-defined glass transition and crystallization temperatures.

The optical measurements revealed electronic transitions associated with transition metal ions, resulting in color variations and band gap values between 1.75 and 2.05 eV for the GMn3, GMn5, and GMn10 samples (not determined for GMn1). Magnetic measurements indicated interactions between Fe^3+^ and Mn^2+^ ions, with *g*-factors close to 2.0 and line width broadening, suggesting the presence of magnetic interactions and clusters or nanoparticles embedded in the amorphous matrix. Furthermore, the hysteresis curves showed an increase in magnetization and coercivity with increasing manganese concentration, indicating the evolution of the magnetic behavior of the system with contributions from asperomagnetic and superparamagnetic-like regimes.

Overall, the results demonstrate that manganese ore incorporation enables the simultaneous tuning of the structural, optical, and magnetic properties of calcium aluminosilicate glasses derived from mining tailings, highlighting their potential for specific applications such as low-field magnetic sensors and optical devices based on absorption and color control, particularly in systems where tunable multifunctional properties are required. Furthermore, this study reinforces the potential of reusing mineral tailings as a sustainable route for the development of functional glass materials.

## Supplementary Material



## Notes

XRD patterns with phase identification of the precursors (scheelite tailings and manganese ore) (PDF)
