# Supplementary material for: Natural Manganese Ore as a Functional Additive in Calcium Aluminosilicate Glasses
Source: ACS Omega. 2026 Aug 21;11(34):51055–66. doi: 10.1021/acsomega.6c02999 (PMC13625113; doi:10.1021/acsomega.6c02999)

Natural Manganese Ore as Functional Additive in Calcium Aluminosilicate Glasses

José G. O. Silva[1], Rai F. Jucá[2], Israel F. Cardoso*[1], Adriana P. B. dos Santos[3], and João M. Soares[1]

[1]Department of Physics, State University of Rio Grande do Norte, 59625-620, Mossoró, RN, Brazil
[2]Department of Physics, Federal University of Ceará, 60021-970, Fortaleza, CE, Brazil
[3]Department of Chemistry, State University of Rio Grande do Norte, 59625-620, Mossoró, RN, Brazil

*Email: israel20231006114@alu.uern.br

Fig S1: XRD measurement and phase identification for scheelite tailing precursor:

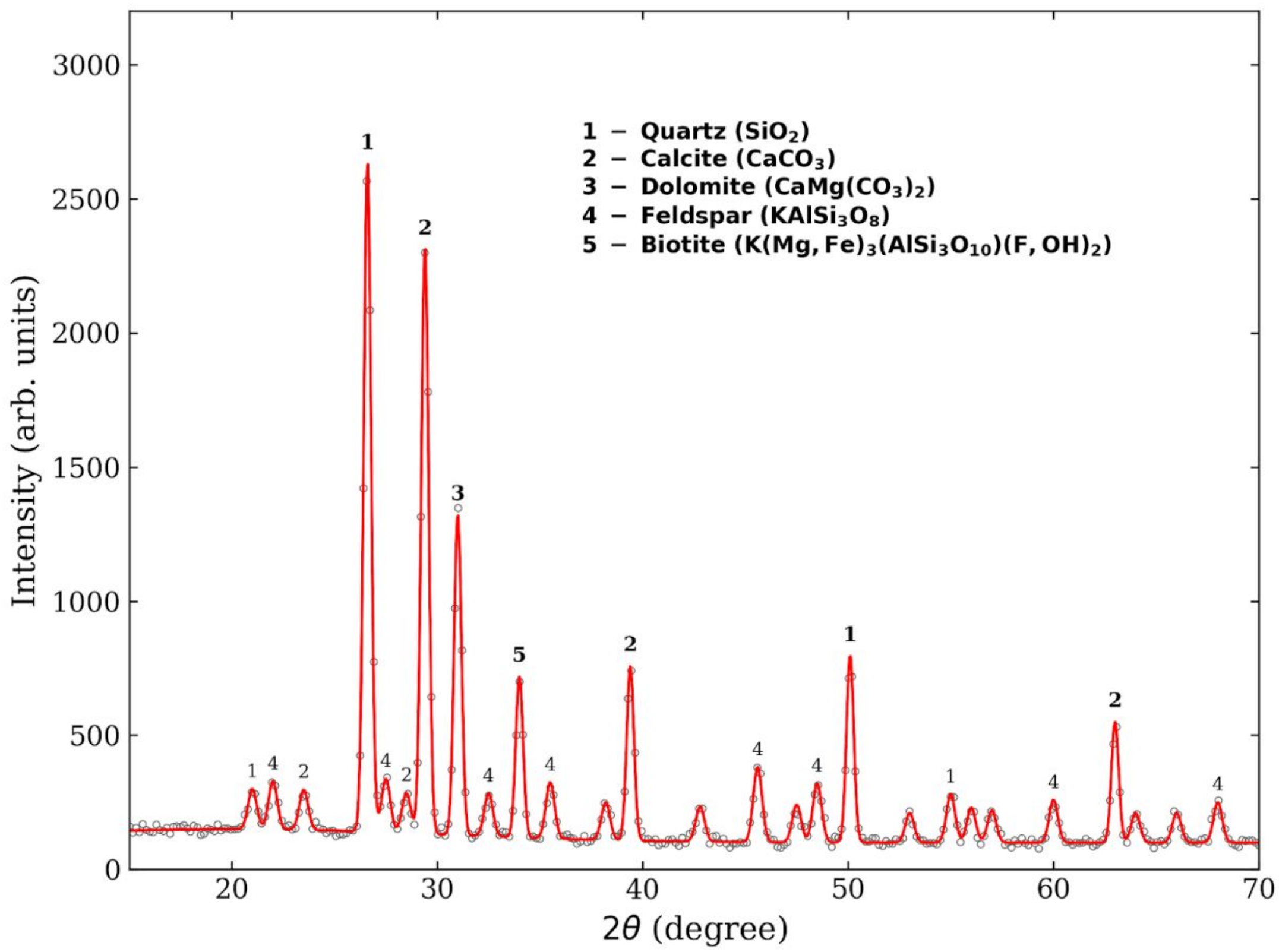

Fig S2: XRD measurement and phase identification for manganese ore precursor:

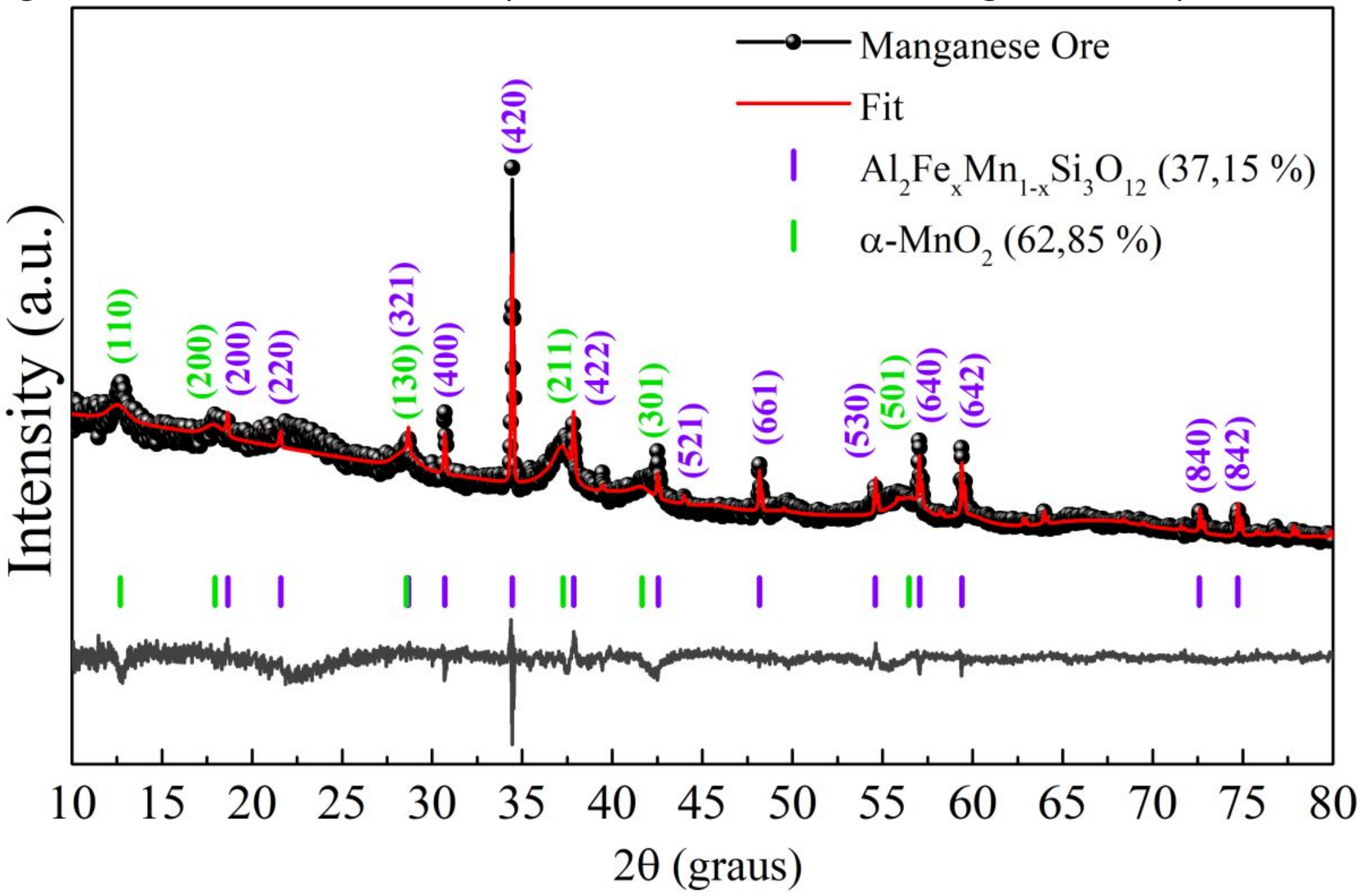

Supplement: Supplementary file 1 [file ao6c02999_si_001.pdf]
